# Traffic safety at level crossings in the Czech Republic

**DOI:** 10.1016/j.heliyon.2024.e39739

**Published:** 2024-10-29

**Authors:** Jan Strohmandl, Pavel Tomášek, Miroslav Tomek, Dušan Vičar, Jakub Rak, Roman Novák

**Affiliations:** Tomas Bata University in Zlín, Nám. T. G. Masaryka 5555, 760 01 Zlín, Czech Republic

**Keywords:** Accident, Emergency event, Level crossing, Railway, Safety, Transport

## Abstract

The article deals with the issue of evaluation of the causes of accidents at level crossings in the Czech Republic, including the prediction of the development and proposals of measures for prevention and thus reduction of their number. The goal of this work is to verify the hypothesis that the number of accidents at level crossings in the Czech Republic is decreasing. The authors use available data from the Ministry of Transport and process statistical data for ten years (2013–2022) of railway operation. Accidents are divided into groups related to accidents at level crossings and the result is expressed in graphical form. These data are then processed and evaluated using the method of regression data analysis. The results of the work are also new proposals and recommended measures to reduce the accident rate at level crossings.

## Highlights

• Railway emergency events are rising.
• Level crossing incidents with just a warning cross are on the decline.
• Proposed measures could cut down collisions.
• By 2022's end, 9355 km of railway had 7646 crossings.

## 1 Introduction

Rail transport is a form of public transport that serves society, with the aim of transporting passengers and freight safely, quickly, punctually, reliably, and economically [1]. This transport is carried out by means of railway transport on the railway line. An inseparable part of railway lines is fixed installations designed to ensure the safety and smoothness of rail transport [2]. Transport structures such as tunnels, bridges, culverts can also be included in this entire railway infrastructure [3].

Railway transport in the Czech Republic has its beginnings in the first half of the 19th century. After the collapse of the Austro-Hungarian Empire, this transport was taken over by the Czechoslovak Republic. Throughout the existence of railways in the Czech lands, the state was in most cases the majority owner, but the railway network was built by private entities and investors. During that time, the railway has undergone many modernizations. In the 1950s and 1960s, the largest and most important development of the railway was the introduction of electric traction, which electrified a significant part of strategically important national and international sections. Unfortunately, in the past, it was not possible to electrify the entire railway network on the territory of the former Czechoslovakia, and so this electrification is still awaited on many sections [4]. The length of the railway network in the Czech Republic as of December 31, 2012 was 9459 km [5], and as of December 31, 2022 it was 9355 km [6].

The dense railway network in the Czech Republic has a negative impact, as with the increase in road and rail transport, there are many emergency events, including at level crossings, which also bring death or injury to persons and significant damage to property.

The number of level crossings in the Czech Republic, although decreasing every year, is one of the largest in Europe [6]. These level crossings of the railway with roads must be secured in the prescribed manner and must comply with the conditions of the relevant laws, standards, and regulations. Even if the level crossing meets these conditions, the occurrence of emergency events is not excluded. Most emergency events arise due to the cause on the part of road users, who unnecessarily risk their lives with lives of other users of the level crossing by their irresponsible behaviour.

A precondition for effective prevention from the point of view of safety at level crossings is the fulfilment of all requirements stipulated by legal regulations, technical standards and recommendations of the Rail Authority and the Police of the Czech Republic. The most important legal regulations in the field of safety at level crossings include Railways Act [7], Road Traffic Act [8] and Decree of the Ministry of Transport issuing the railway transport regulations [9].

A level crossing is a place where a railway crosses another type of road [7]. From a historical point of view, the railway and road level crossing were created mainly at a time when the growing road infrastructure began to intersect the railway lines on which in the 19th century mined coal and aggregates were mainly transported to the places of final use [10]. According to Ref. [7], a level crossing is defined as a place where: the level of the road crosses with the railway or with another track lying on a separate body and marked with the appropriate traffic sign.

According to valid legal standards, railway crossings in the Czech Republic must be protected, i.e. secured for road users.

As of December 31, 2022, there were a total of 7646 level crossings and level crossings on railway lines with the right to manage by the Railway Administration in the Czech Republic (Table 1), of which almost half (3,377) were secured only by a warning cross, i.e. 44.2 %. Over the past 10 years, there has been a significant decrease in the number of level crossings, as of December 31, 2012 the Railway Infrastructure Administration (the predecessor of the current Railway Administration) registered a total of 8095 level crossings. It follows from the above that in 10 years the number of level crossings decreased by 449. Despite this, the density of level crossings in the Czech Republic is high and represents an average of 0.82 level crossings per 1 km of railway. Table 1 represents not only information about selected country but also contains comparison with other European countries. From this perspective the Czech Republic belongs to the top countries with the highest density of level crossings. The traffic performance is presented in Table 2. In general, the traffic volumes reflect the population of each country. Interesting ratio of passenger transport by rail/road is in Switzerland where 18 % of all passenger-km is in favor of the railway. This number is usually much lower in other countries (for instance 10 % in the Czech Republic).

**Table 1:** – Basic overview of level crossings in the Czech Republic neighbouring and selected other European countries in the year of 2022 (or using the latest data available) according to level crossing (shortly as LC) safety equipment [11,[28], [29], [30]], sorted by density of LCs.

Country	LC secured only by a warning cross (passive)	LC secured by level crossing safety system (active)	Total number of LCs	Surface area (km^2^)	LCs per 100 km^2^
**Switzerland (year 2021)**	1294	3059	4353	41,285	**10.54**
**Czech Republic**	3377	4269	7646	78,871	**9.69**
**Hungary**	3194	2549	5743	93,012	**6.17**
**Netherlands**	570	1682	2252	37,378	**6.02**
**Germany**	5863	10,012	15,875	357,569	**4.44**
**Slovak Republic (year 2018)**	(not available)	(not available)	2088	49,035	**4.26**
**Poland**	6402	5430	13,272	311,928	**4.25**
**Austria**	1417	1567	2984	83,878	**3.56**
**Slovenia**	347	341	688	20,273	**3.39**
**France (year 2020)**	5061	12,396	17,457	638,475	**2.73**
**Italy**	489	3646	4135	302,079	**1.37**
**Sweden**	2900	2886	5786	447,424	**1.29**

**Table 2:** – Basic overview of traffic volumes in the Czech Republic neighbouring and selected other European countries in the year of 2022 (or using the latest data available, written next to the value in parentheses) [[38], [39], [40], [41], [42]], sorted alphabetically.

Country	Passenger transport by rail (mill. passenger-km)	Passenger car transport by vehicles registered in the country (mill. passenger-km)	Goods transport by rail (mill. tonne-km)	Goods transport by road by vehicles registered in the country (mill. tonne-km)
**Austria**	(2018) 13,205	(2018) 78,545	21,980	26,831
**Czech Republic**	9823	91,245	16,697	65,794
**France**	102,814	809,404	35,282	173,353
**Germany**	94,630	840,834	123,067	303,948
**Hungary**	7817	72,570	11,350	37,444
**Italy**	46,142	(2018) 722,894	24,130	151,100
**Netherlands**	17,105	(2018) 144,700	7119	67,148
**Poland**	23,527	(2018) 212,416	59,032	385,088
**Slovak Republic**	3338	(2018) 28,460	7254	31,488
**Slovenia**	645	23,420	4084	24,308
**Sweden**	(2021) 8028	(2018) 116,000	(2021) 23,449	47,865
**Switzerland**	19,340	85,800	12,136	17,400

In places where there is a level crossing of road and rail transport, level crossing safety installations are used to ensure the safety of road and railway traffic. These level crossing devices can be divided according to their safety.

• level crossing with a warning cross only,
• level crossing with light signalling,
• level crossing with mechanical protection.

For information, the database of the Traffic Police Service Directorate of the Police Presidium of the Czech Republic distinguishes railway crossings as follows [12].

• technically secured by level crossing safety equipment,
• not technically secured or secured only by a warning cross.

Level crossing safety shall clearly, timely and unambiguously warn road users in the event that a railway vehicle is approaching the level crossing on the track and must be given way by road users. Road users are warned by means of a level crossing safety warning system. The warning is signalled in such a way that even a slow and long road vehicle, which cannot stop safely before the level crossing, reliably leaves the level crossing before the barriers are activated, if the level crossing is equipped with them (alternatively, if the road vehicle has passed the border of the danger zone on the other side of the crossing). The signalling of the warning must be ensured in such a way that it restricts traffic on the road only for the necessary time to ensure the safe passage of railway rolling stock [13].

With the use of regression analysis, the following hypothesis will be verified: “*During the last 10 years (2013–2022), the number of accidents at level crossings in the Czech Republic has been decreasing*”. The analysis will be based on the number of emergency events on national and regional railways and sidings between 2013 and 2022 (hereinafter referred to as the “monitored period”). The defined statistical features (*SZi*) of the studied statistical units in this area are statistically dependent.

Subsequently, within the framework of linear regression analysis, partial objectives will be verified, which will be used to evaluate individual types of emergency events in the range of the monitored period (i.e. 10 years) and will consist of the evaluation of these individual types when examining the number of collisions (accidents) at level crossings equipped with.

• warning cross,
• light and sound security signalling,
• light and sound security signalling with barriers.

Part of this article is also the specification of selected road transport participants on the number of accidents at level crossings and, last but not least, proposals for their minimization.

The chosen 10-year period may be viewed as a relatively small sample size, which may not sufficiently support the assumptions of linear regression. The official resources available provide only a limited history of detailed data relevant to this article. Nevertheless, the authors sought to identify at least some potential trends and patterns.

The aim of this article is to use regression analysis (using both annual and monthly data) to verify the hypothesis, including new proposals for increasing the safety of railway crossings.

The issue of traffic accidents at level crossings has been addressed by many authors in various parts of the world, where e.g. the work in Ref. [14] is based on data in a period of 20 years, published on Wikipedia for the European Union. Moreover, the security of level crossings and corresponding optimization of road-rail infrastructure was solved within a European project called SAFER-LC [35] with many published results (for instance evidence from the SAFER-LC project in Italy [36]) accompanied with a toolbox to make level crossings safer [37]. The issue of accident factors in Argentina is dealt with in Ref. [15]. Risk assessment in railway transport safety was carried out in Ref. [16]. The issue of accidents at railway stations is dealt with in the work [17]. The causes of railway accidents in Bangladesh are discussed in Ref. [18], in Slovakia, the issue was addressed in Ref. [19]. The influence of the number of level crossings on train delays (including possible accidents on them) is among others discussed in Ref. [27]. A master's thesis from 2017 called Accidents on the railways and its prevention [32] analyses the safety on railways in the Czech Republic and which is also focused on a problem concerning transport education teaching with orientation on prevention connected with people moving on railways at the chosen primary school. Risk assessment study and improving railway safety is the goal of a dissertation from 2013 [33]. Another doctoral thesis from 2002 is aimed at railway safety in relation with risks and economics in general [34].

Unlike the above-mentioned authors, the authors of this article deal with specific types of accidents and are based on relevant data from the official sources of the Ministry of Transport of the Czech Republic.

## 2 Materials and methods

Many emergency events occur on the railway every year. Emergency events at level crossings are also an integral part of them.

Pursuant to Act No. 266/1994 Coll., on Railways, an extraordinary event is defined as an accident or incident that occurred in connection with the operation of rail transport or the movement of a railway vehicle on the track or within the railway perimeter and that endangered or disrupted the safety, regularity and continuity of rail transport operation, the safety of persons and the safe function of buildings and equipment, or endangers the environment [7].

### 2.1 Overview of emergency events on railways and level crossings in 2013-2022

**Fig. 1:**
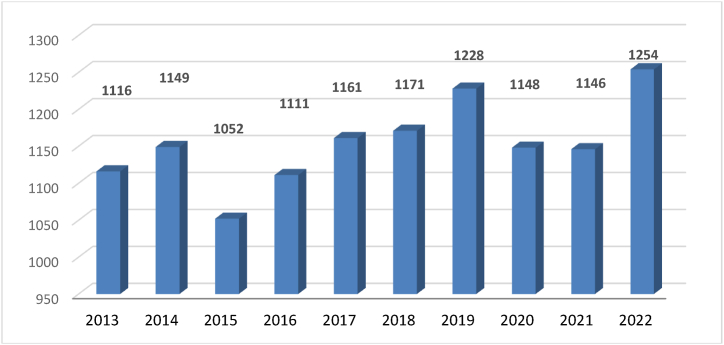
– Statistics of the number of emergency events on railway lines in the Czech Republic [11].

During the period under review, a total of 11,536 events occurred on railway lines (Fig. 1) (excluding the special railway – subway). The largest number of 1254 emergency events occurred in 2022 and the smallest 1052 emergency events occurred in 2015.

**Fig. 2:**
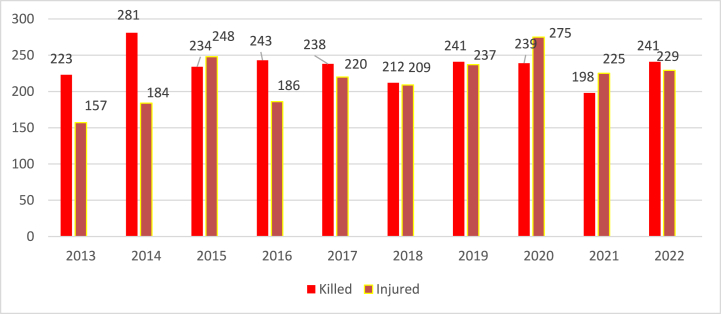
– Statistics of the number of killed and injured on railway lines in the Czech Republic [11].

One of the accompanying phenomena of emergency events on the railway has been, is and will be accidents, which can be characterized as an event resulting in death, bodily injury, or other harm. A total of 2350 persons were killed and 2170 persons injured in these accidents on railway lines (except for the special railway – subway) during the period under review (Fig. 2).

In the case of railways in the Czech Republic, it can be stated that on average 20 persons were killed and approximately 18 persons were injured per month during the period under review. The highest number of persons killed was in 2014 and the highest number of injured persons was in 2020. Every year, the largest share in the number of killed persons in the statistical outputs of the Rail Inspection is occupied by collisions between trains and people, which can also include collisions at level crossings.

**Fig. 3:**
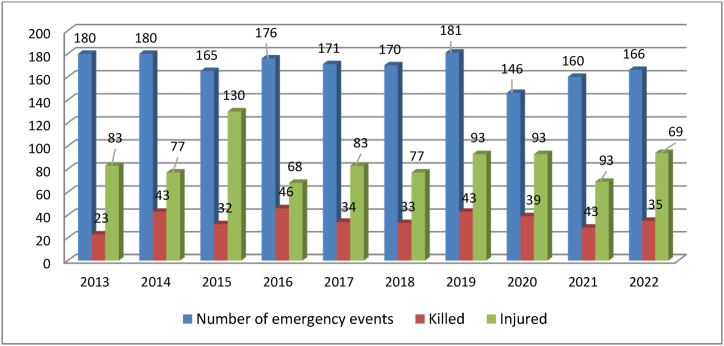
– Statistics of the emergency events and their consequences at level crossings in the Czech Republic [11].

Over the 10-year period, 1695 emergency events occurred at level crossings in the Czech Republic, during which a total of 353 persons were killed and 868 persons injured (Fig. 3). It follows from the above that on average about 36 persons are killed and 87 injured at level crossings per year. The highest number of killed persons was in 2016 (46 persons) and the lowest in 2013 (23 persons). The highest number of injured was in 2015 (130 persons) and the lowest was in 2016 (68 persons). Unfortunately, in recent years, the number of injured has been relatively high despite the measures taken by the railways.

Interesting data also emerge from the overview of emergency events for the monitored period and their consequences on persons at individual level crossings in terms of mortality and type of injury (Table 3). The overview shows that from the point of view of minimizing collisions of persons at individual level crossings, a level crossing secured by light signalling with barriers seems to be the most advantageous, but surprisingly not from the point of view of mortality and injuries of persons. In this type of level crossing, there is the worst ratio of killed people per collision (0.50, considering the total numbers from the statistics of the selected 10 years). Level crossings secured by security signalling without barriers has lower ratio (0.23). Moreover, level crossings equipped only with warning crosses has the lowest ratio of killed per collision (0.08). Further analysis of this situation is not the aim of this article.

**Table 3:** – Number of collisions of persons (emergency events) in the Czech Republic at individual level crossings according to their safety [5].

Year	Warning crosses			Light security signalling			Light security signalling with barriers			∑		
	Number of collisions	Killed	Injured	Number of collisions	Killed	Injured	Number of collisions	Killed	Injured	Number of collisions	Killed	Injured
**2013**	81	4	25	83	12	44	16	7	14	180	24	83
**2014**	71	5	28	84	25	45	25	13	3	180	43	76
**2015**	70	8	19	71	13	81	24	11	30	165	32	130
**2016**	69	7	21	78	25	34	29	14	13	176	46	68
**2017**	72	4	28	75	17	51	22	12	4	171	33	83
**2018**	66	3	16	77	20	49	27	10	12	170	33	77
**2019**	63	4	29	98	25	57	20	12	9	181	41	95
**2020**	57	11	38	61	13	45	28	15	10	146	39	93
**2021**	56	2	21	83	16	45	21	11	3	160	29	69
**2022**	53	5	18	85	15	72	27	14	4	166	34	94
**∑**	**658**	**53**	**243**	**795**	**181**	**523**	**239**	**119**	**102**	**1695**	**353**	**868**

Based on the data analysis, it can be stated that the largest number of emergency events at level crossings, as already stated, occurred in 2019 (181). This year, 41 people were killed and 95 people were injured. On the other hand, the lowest number of emergency events occurred in 2020 (146). One of the reasons of the lower number may be the case of the restriction on the free movement of people due to the government-approved measures against the spread of coronavirus.

Although this is the lowest number of emergency events in the monitored period this year, the consequences were quite tragic, with 39 people killed and another 93 people injured. From the point of view of killed persons, the year 2016 can be considered the most tragic, when 46 persons died at level crossings. The lowest number of deaths was recorded in 2013 (23 persons).

Collisions of passenger trains with lorries play a significant role, especially accidents with a larger number of injured persons in 2015 (e.g. March 24, 2015, Obrataň – 12 injured and damage 7,878,862 CZK; May 25, 2015, Velké Pavlovice – 19 injured and damage CZK 3,400,409; last but not least, on July 22, 2015 in Studénka – 25 injured, 3 persons killed and damage 156,700,000 CZK) [11].

From the point of view of long-term statistical data, considering absolute values, the most tragic impacts are collisions at level crossings secured by light signalling without barriers. The largest number of 98 emergency events was in 2019. The highest number of persons killed on this type of level crossing safety took place this year, namely 25 (the same as in 2014 and 2016). The highest number of injured was in 2015 (81 persons) and the lowest number of injured persons was in 2013 (44 persons).

The second largest group in terms of the number of emergency events and injured persons consists of level crossings equipped only with a warning cross, which can be classified as the lowest level crossing safety. The highest number of emergency events occurred in 2013 (81). The highest number of deaths was recorded in 2020 (11 persons) with a lower number of emergency events (57). The figures show that the number of emergency events at these level crossings has been decreasing since 2021. This may relate to the addition of warning crosses at level crossing light safety installations.

Railway crossings equipped with light signalling in combination with barriers seem to be the safest type of level crossing in terms of the number of emergency events, killed and injured. However, there are still many of these emergency events. The highest number of collisions was recorded in 2016 (29), the highest number of killed was recorded in 2020 (15 persons). The most serious emergency events of that year can be considered emergency events, which happened on April 27, 2020, when a passenger train collided with a passenger car at a level crossing on the line Heřmanova Huť – Nýřany. This emergency event claimed 4 victims. The highest number of injured persons at these level crossings was in 2015 (30 persons).

An overwhelming number of emergency events at level crossings every year are caused by unruly road users (Table 4), who fundamentally do not respect the relevant legal norm [8]. Drivers of all types of vehicles most often commit offences by ignoring the light and sound signalling of the level crossing safety installation, or when they are not sufficiently convinced whether a train is approaching the crossing.

**Table 4:** – Collisions at level crossings by road users in the Czech Republic [11] (EEs stands for the number of emergency events).

		2013	2014	2015	2016	2017	2018	2019	2020	2021	2022
**Bus**	EEs	1	2	1	1	1	0	0	0	0	1
	Killed	0	0	0	0	0	0	0	0	0	0
	Injured	2	5	0	1	0	0	0	0	0	2
**Cyclist**	EEs	4	4	11	4	7	4	5	1	5	4
	Killed	2	2	5	0	3	2	3	1	2	2
	Injured	0	1	2	3	3	1	1	0	2	1
**Motorcycle**	EEs	0	2	2	2	0	3	3	2	3	2
	Killed	0	1	0	0	0	3	2	2	0	0
	Injured	0	1	1	1	0	0	1	0	3	1
**Lorry**	EEs	20	15	21	17	28	17	25	20	22	18
	Killed	0	0	3	2	0	1	1	0	2	2
	Injured	16	6	72	4	22	6	32	20	10	18
**Person**	EEs	17	29	16	30	23	18	29	25	15	26
	Killed	11	20	10	24	18	13	24	19	15	21
	Injured	5	8	6	7	5	5	5	6	0	5
**Passenger car**	EEs	125	120	108	114	111	116	115	94	111	112
	Killed	11	18	14	19	13	14	13	17	10	9
	Injured	42	48	44	42	53	51	54	51	54	58
**Tractor**	EEs	13	3	4	5	1	9	4	3	4	3
	Killed	0	0	0	1	0	0	0	0	0	1
	Injured	8	0	5	9	0	11	0	5	0	9

Bus accidents at level crossings are not so frequent, but until 2018 there was on average at least one such case every year [11]. The fact that from 2018 to 2021 there were no emergency incidents at a level crossing in connection with a bus or a railway vehicle and no person was killed during the monitored period can be considered positive. In recent years, it has been quite common in the Czech Republic to encounter a situation in which truck drivers do not respect the level crossing safety lights that have been triggered. After lowering the barriers, these drivers are trapped at the level crossing and often do not know what to do. This scenario was also a consequence of the highest number of killed and injured in 2015. Although this year was far from being one of the most collisions per year, it has been one of the most serious in the last 10 years thanks to the accident in Studénka. As mentioned earlier, this accident occurred on July 22, 2015 at a level crossing equipped with a light level crossing safety installation with barriers [11].

Motorcyclists are not exempt from accidents at level crossings either. The highest number of emergency events in the monitored period occurred in 2018, when 3 motorcyclists were killed in a total of three collisions at level crossings.

In the case of cyclists and pedestrians, the common cause of their death is knowingly bypassing or crawling under lowered barriers. In these situations, unlike level crossings equipped only with traffic lights, it is not possible to speak of oversight or mistake on the part of the participants of emergency events, but of conscious risk-taking and disregard of legal regulations. These cases almost always end in death. The worst year can be considered 2015, when there were 11 emergency events, during which 5 cyclists died and another 2 cyclists were injured. As far as pedestrians are concerned, the highest number of pedestrians was killed in 2016 and 2019, when 24 persons were killed at level crossings.

Even more detailed data are publicly available. Table 5 contains monthly data of numbers of emergency events, killed and injured. However, the information about security equipment of level crossing in these cases are missing in the reports.

**Table 5:** – Collisions at level crossings in months in the Czech Republic [11] (EEs stands for the number of emergency events).

		2013	2014	2015	2016	2017	2018	2019	2020	2021	2022
**January**	EEs	18	11	14	13	25	20	22	11	17	13
	Killed	3	3	1	7	6	4	2	1	5	1
	Injured	3	8	2	5	8	11	3	6	2	5
**February**	EEs	17	15	9	15	4	15	11	8	17	9
	Killed	3	6	1	0	0	3	0	1	0	2
	Injured	4	4	7	2	3	6	3	14	10	4
**March**	EEs	6	8	8	20	8	9	11	7	12	9
	Killed	3	2	1	9	2	2	2	2	7	2
	Injured	1	3	17	10	3	4	6	4	4	4
**April**	EEs	16	17	10	11	13	11	18	9	9	18
	Killed	1	5	2	2	3	2	4	6	3	4
	Injured	9	6	7	1	12	8	8	4	2	7
**May**	EEs	14	17	14	15	17	10	18	11	11	21
	Killed	3	1	2	2	2	2	4	4	1	4
	Injured	6	10	23	7	5	2	3	5	9	11
**June**	EEs	13	19	23	8	11	17	13	13	12	11
	Killed	3	3	2	1	3	2	1	4	0	1
	Injured	6	11	8	5	0	5	6	19	3	5
**July**	EEs	22	18	17	18	17	15	24	26	19	15
	Killed	2	5	3	5	5	5	10	7	2	6
	Injured	23	8	32	6	8	13	27	10	11	15
**August**	EEs	20	14	15	20	15	22	16	12	14	15
	Killed	1	5	4	6	7	1	3	4	0	2
	Injured	3	7	9	6	3	9	15	3	10	10
**September**	EEs	15	20	19	16	19	11	10	15	9	15
	Killed	1	3	6	4	2	2	3	4	5	2
	Injured	11	6	13	13	18	3	9	13	2	6
**October**	EEs	13	16	10	21	15	10	17	8	16	16
	Killed	1	2	2	5	2	1	7	3	3	6
	Injured	6	5	8	7	4	3	8	4	7	13
**November**	EEs	11	12	13	11	15	15	15	12	10	10
	Killed	0	6	2	1	2	4	5	0	1	2
	Injured	5	2	2	2	12	5	2	6	3	8
**December**	EEs	15	13	13	8	12	15	6	14	14	14
	Killed	3	2	6	4	0	5	2	3	2	3
	Injured	6	7	2	4	7	8	3	5	6	6

### 2.2 Regression analysis for trend identification

Linear regression analysis was used to verify the hypothesis defined in introduction (*"During the last 10 years (2013–2022), the number of accidents at level crossings in the Czech Republic has been decreasing”*). Regression analysis can be used to describe the dependence of the observed dependent quantitative variable on one or more other independent quantitative variables or categorical variables converted into auxiliary so-called “artificial” variables [20]. In the case of trend estimation, the observed variable acts as the output variable *y* and the input variable *x* is the relevant period *t*. The aim of the regression is to find the dependence under investigation using an appropriate regression model and equation. The most commonly used model is a linear model described by a straight line or by polynomial functions. If a categorical variable enters the model, such as the seasonal component of the model, it must be recoded into auxiliary variables, most often “dummy” variables are used. These are zero-one variables, in number, according to the number of categories of the original variable. In the case of monthly data, this is 12. One less dummy then enters the model, as the omitted one represents the reference category and against which the resulting regression coefficient is related and compared. A good regression model contains variables with significant regression coefficients and at the same time the overall model is significant according to the F-test at the chosen significance level α, most commonly 5 % or 10 %. A good model is also recognised by the proportion of explained variability by the model being as high as possible. This is indicated by the coefficient of determination *R*^*2*^. Once a suitable regression model describing the phenomenon is found, the regression equation can be estimated, and the identified trend applied [31]. However, the trend estimate is very approximate, and its informative value is not very high. For more accurate estimates, it would be necessary to have a model with a higher number of input variables that have a significant effect on the variable being modelled. These are the environmental conditions affecting the phenomenon. Therefore, prediction based on previous values is very tentative. Such a limited trend model does not respect some of the limitations of the observed variable. For example, the values of variables in the most recent years preceding the forecast year do not have a significantly higher weight in the model than older data from the years at the beginning of the forecast period. Such a simplified model is very limited and therefore its prediction reflects the historical development of the variable rather than the current conditions for the future development of the variable. Therefore, such a model is at most suitable for estimating the trend for only one subsequent period (one more year), but not for estimating the long-term trend. For the following years, it would be advisable at least to use the already extended data set for the next past period (year) and to find again a suitable quality regression model on the updated data and thus identify a new current trend.

A linear regression line can be expressed by:

(1) $$y=b_{1}x+b_{0}$$

where: *b*_*1*_ – slope of a straight line,

*b*_*0*_ – intercept (*y*-axis displacement) [21].

The regression lines can be solved by calculating a system of equations [21]:

(2) $$\sum s_{i}=kb_{0}+b_{1}\sum x_{1}$$

(3) $$\sum s_{i}x_{i}=b_{0}\sum x_{1}+b_{1}\sum x_{1}^{2}$$

The application of the chosen method of regression analysis in this study is the following. The values of the statistical characters *SZ-1* were entered on the *x*-axis (it is the examined period of 10 years; therefore, the elements *x*_*i*_ represent the years from 2013 to 2022) and the statistical characters *SZ*_*i*_*-2* on the *y*-axis (the real numbers of emergency events in the corresponding years). Microsoft Excel has been used for the purpose of the regression analysis.

The most important value in this statistical approach is the *p* value, which contains information whether the hypothesis can be statistically proven. The value of 0.05 is considered to be borderline, i.e. if the *p* value is lower than 0.05, it can be considered as a statistically demonstrable tendency of decrease or increase in the number of traffic accidents at level crossings during the monitored period.

To verify the hypothesis, regression analysis was applied to address the number of emergency events – collisions (*s*_*i*_).

• at level crossings in general (*SZ*_*1*_*-2*) and subsequently also analysis of a subset of emergency events at level crossings equipped with:
  o warning cross (*SZ*_*2*_*-2*),
  o light and sound security signalling (*SZ*_*3*_*-2*),
  o light and sound security signalling with barriers (*SZ*_*4*_*-2*),
• on the railroad (*SZ*_*5*_*-2*).

## 3 Results

From the development of the selected ten years, it can be stated that the number of emergency events at level crossings is relatively stable, while the reduction of unfavourable numbers of these accidents is possible in various ways. The obtained statistics show that every fifth accident causes a human death. The vast majority of accidents at level crossings continue to be caused by unruly road users who violate the provisions [8]. The most frequent offence is ignoring the light and sound signalling of the level crossing safety equipment, not making sure whether a train is approaching the level crossing, bypassing or crawling under lowered barriers.

The input data resulting from the preceding figures and tables are summarized in Table 6.

**Table 6:** – Summarized input data (numbers of various kinds of emergency events in the Czech Republic) for further regression analysis.

SZ-1 (year)	SZ_1_-2 (emergency events at level crossings in general)	SZ_2_-2 (emergency events at level crossings equipped with warning cross)	SZ_3_-2 (emergency events at level crossings equipped with light and sound security signalling)	SZ_4_-2 (emergency events at level crossings equipped with light and sound security signalling with barriers)	SZ_5_-2 (emergency events on the railroad)
**2013**	180	81	83	16	1116
**2014**	180	71	84	25	1149
**2015**	165	70	71	24	1052
**2016**	176	69	78	29	1111
**2017**	171	73	75	22	1161
**2018**	170	66	77	27	1171
**2019**	181	63	98	20	1228
**2020**	146	57	61	28	1148
**2021**	160	56	83	21	1146
**2022**	166	53	85	27	1254

**Fig. 4:**
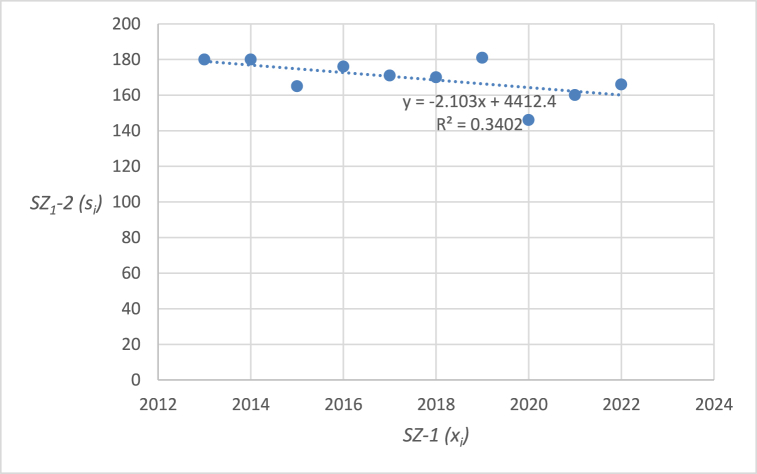
– Regression analysis of the statistics of the emergency events at level crossings in the Czech Republic.

**Fig. 5:**
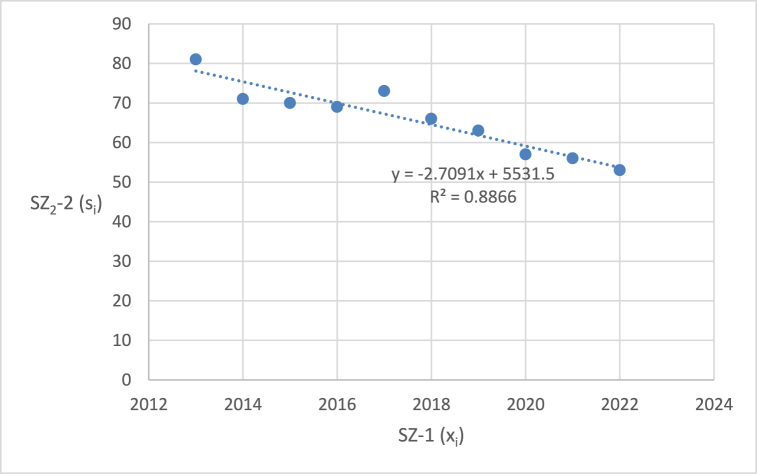
– Regression analysis of the statistics of emergency events at level crossings equipped only with a warning cross in the Czech Republic.

**Fig. 6:**
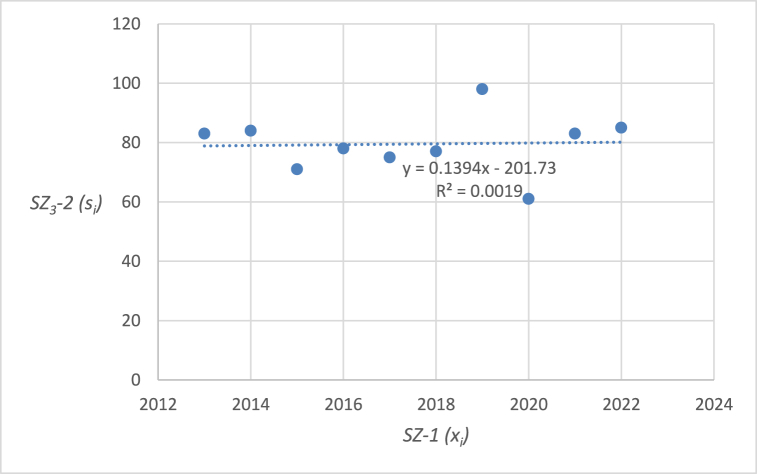
– Regression analysis of the statistics of emergency events at level crossings equipped with light and sound signalling equipment (without barriers) in the Czech Republic.

**Fig. 7:**
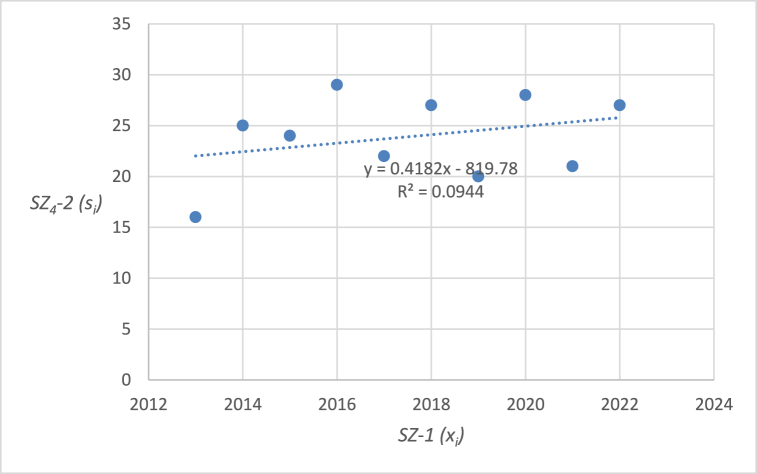
– Regression analysis of the statistics of emergency events at level crossings equipped with light and sound signalling equipment with barriers in the Czech Republic.

**Fig. 8:**
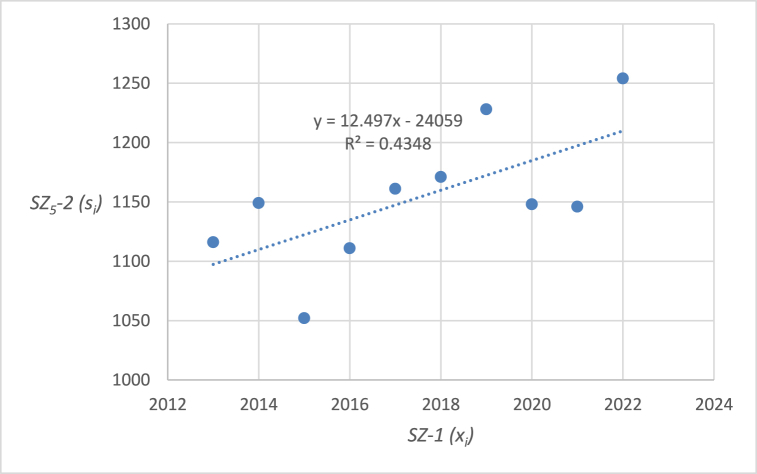
– Regression analysis of the statistics of emergency events on the railway in the Czech Republic.

The graphical representation of the linear regression analysis, including the determination of the equation of the regression line and the reliability value of selected emergency events related to level crossings in the Czech Republic are shown in Fig. 4, Fig. 5, Fig. 6, Fig. 7, Fig. 8. Fig. 4 represents the statistics of the emergency events at level crossings in general. Fig. 5 depicts the statistics of the emergency events at level crossings equipped only with a warning cross. Fig. 6 shows the statistics of the emergency events at level crossings equipped with light and sound signalling equipment (without barriers). Fig. 7 shows the statistics of the emergency events at level crossings equipped with light and sound signalling equipment with barriers. Finally, Fig. 8 depicts the statistics of the emergency events on the railway.

The output of the linear regression analysis for the development of the number of emergency events at level crossings (*SZ*_*1*_*-2*) is the equation of the regression line: *y* = −2.103*x* + 4412.4 (*y* represents the number of events in a chosen year, *x*). From a purely statistical point of view, it can be stated that the number of collisions in this category has been decreasing annually in recent years by an average of 2.103 (*b*_*1*_). The prediction of emergency events in 2023 is 158 and in 2024 it is 156 events. Another important output for better information is the correlation coefficient, where its value is −0.5833 and the index of determination is 0.3402.

In the following parts of the article individual partial emergency events are dealt with in a similar way, the results are summarized in Table 7.

**Table 7:** – Calculated parameters of the processed regression analyses by the category of emergency event.

		Regression line equation	Correlation coefficient	Index of determina-tion (*R*^*2*^)	*F* statistics	*p* value
EE at level crossings in total (*SZ*_*1*_*-2*)		*y* = −2.103*x* + 4412.4	−0.5833	0.3402	4.1251	0.07673
Railway crossing signalling	Warning cross (*SZ*_*2*_*-2*)	*y* = −2.7091*x* + 5531.5	−0.9416	0.8866	62.5674	0.00005
	Light and sound security signalling (*SZ*_*3*_*-2*)	*y* = 0.1394*x* – 201.7	0.0436	0.0019	0.0149	0.90576
	Light and sound security signalling with barriers (*SZ*_*4*_*-2*)	*y* = 0.4182*x* – 819.8	0.3072	0.0944	0.8335	0.38795
EE on railway lines (*SZ*_*5*_*-2*)		*y* = 12.4970*x* – 24059	0.6594	0.4348	6.1537	0.03807

Using the equations in the calculations, specific data were obtained for individual types of emergency events at level crossings (Table 7). The index of determination (*R*^*2*^) reveals that selected linear model is the most suitable for the case of *SZ*_*2*_*-2* (linear trend fits almost from 89 %). The other trends are less linear (*SZ*_*5*_*-2* 43 %, *SZ*_*1*_*-2* 34 %, *SZ*_*4*_*-2* 9 %, and *SZ*_*3*_*-2* even below 1 %).

### 3.1 Verification of the hypothesis

As previously noted, the regression analyses rely on data from the years 2013–2022. Consequently, the significance of the regression analyses is directly related to the limited availability of source data during this period.

The statistically demonstrable trend in the number of emergency events at level crossings was evaluated only in the case of *SZ*_*2*_*-2* (emergency events at level crossings equipped with warning cross is decreasing) and *SZ*_*5*_*-2* (number of emergency events on the railroad is increasing). The *p* value is below 0.05 in both cases. On the other hand, *SZ*_*1*_*-2* (number of emergency events at level crossings in general) also shows a downward trend, which, however, is not statistically significant considering 5 % level of significance (but very close), as the corresponding *p* value is 0.07673. Therefore, the hypothesis in the wording “*During the last 10 years (2013–2022), the number of accidents at level crossings in the Czech Republic has been decreasing*” cannot be statistically confirmed in general but only in the case of *SZ*_*2*_*-2* (and partially in the case of *SZ*_*1*_*-2*). For *SZ*_*3*_*-2* (number of emergency events at level crossings equipped with light and sound security signalling) and *SZ*_*4*_*-2* (number of emergency events at level crossings equipped with light and sound security signalling with barriers) it is not possible to statistically prove a downward trend. It is therefore necessary to propose appropriate measures for high non-decreasing (or only slightly decreasing) numbers of emergency events. As part of the discussion, there are proposals for appropriate measures that should serve to reduce the number of emergency events on national and regional railways and sidings.

### 3.2 Seasonality-adjusted regression model of encounter trend on monthly data

The previous analysis used annual data. The annual reports contain also monthly data, however, in this data there is no information about railway crossing signalling (equipped with warning crosses, light and sound security signalling and/or with barriers). Therefore, the further analysis is considering only emergency events at level crossings in total (*SZ*_*1*_*-2*).

**Fig. 9:**
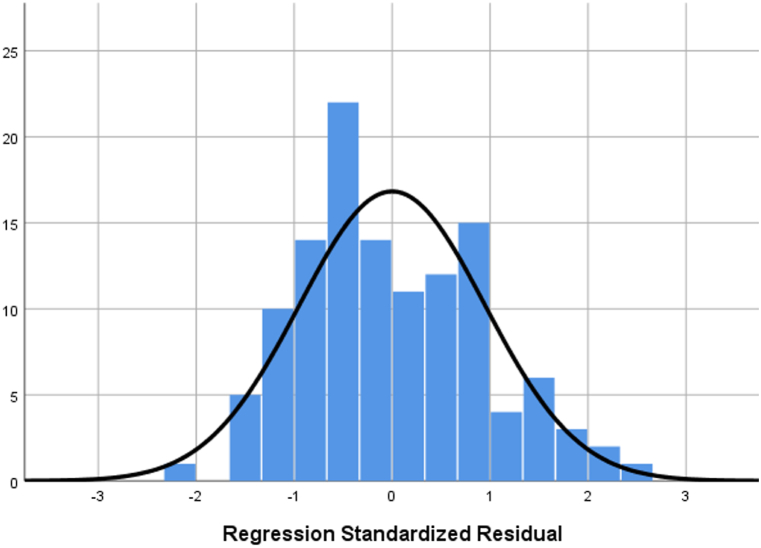
– Histogram of regression standardized residuals of monthly data of emergency events at level crossings in the Czech Republic, 2013–2022 (dependent variable: number of emergency events, mean = 2.09E-16; std. dev. = 0.948; N = 120).

This regression model estimating the monthly trend would explain 31.1 % of the total variability (34.0 % for the annual data). The residuals also satisfy the normality condition, i.e. zero mean and unit standard deviation (histogram in Fig. 9, statistics in Table 8), so this assumption is also satisfied, as with the annual data. The regression coefficients (the constant and some beta coefficients of the seasonal component, namely *s*_*2*_*, s*_*3*_*, s*_*4*_*, s*_*11*_ and *s*_*12*_) are statistically significant according to the T-test at the 10 % significance level (Table 9). While the regression coefficient of the beta for the trend component is not statistically significant, nor are some of the beta coefficients of the seasonal component, since the seasonal component has assumed significance here in the monthly model instead of the trend, seasonality is clearly significant for trend modeling. Moreover, both to track seasonality in the model and to adjust the trend for seasonality, all coefficients in the model are needed. Such a model otherwise seems appropriate for identifying an annual trend adjusted for monthly seasonality.

**Table 8:** – Calculated residuals statistics of monthly data of emergency events at level crossings in the Czech Republic, 2013–2022 (dependent variable: number of emergency events).

	Minimum	Maximum	Mean	Std. Deviation	N
*Predicted Value*	9.01	19.89	14.12	2.405	120
*Residual*	−8.088	9.937	0.000	3.575	120
*Std. Predicted Value*	−2.127	2.397	0.000	1.000	120
*Std. Residual*	−2.145	2.636	0.000	0.948	120

**Table 9:** – Coefficients of the seasonal component of monthly data of emergency events at level crossings in the Czech Republic, 2013–2022 (dependent variable: number of emergency events).

Model	Unstandardized Coefficients		Standardized Coefficients	t	Sig.
	B	Std. Error	Beta		
(constant)	17,364	1362		12,746	0,000
*t*	−0,175	0,120	−0,117	−1462	0,147
*s*_*1*_	0.000				
*s*_*2*_	−4400	1686	−0,283	−2609	0,010
*s*_*3*_	−6600	1686	−0,425	−3914	0,000
*s*_*4*_	−3200	1686	−0,206	−1898	0,060
*s*_*5*_	−1600	1686	−0,103	−0,949	0,345
*s*_*6*_	−2400	1686	−0,155	−1423	0,158
*s*_*7*_	2700	1686	0,174	1601	0,112
*s*_*8*_	−0,100	1686	−0,006	−0,059	0,953
*s*_*9*_	−1500	1686	−0,097	−0,890	0,376
*s*_*10*_	−2200	1686	−0,142	−1305	0,195
*s*_*11*_	−4000	1686	−0,258	−2372	0,019
*s*_*12*_	−4000	1686	−0,258	−2372	0,019

According to the seasonal regression coefficients for individual months (Table 9), the difference in specific months relative to the reference (first month, *m*_*1*_) can be observed. In all other months (except *m*_*7*_), the estimated monthly number of encounters is lower compared to the first month, the lowest number would be in the third month (6.6 encounters less than in the first month).

The values relative to the average month can be estimated after adjusting the individual monthly regression coefficients (and the constant, but not the trend estimate) by the average monthly coefficient. The equation of the regression model containing both the trend and seasonal components then looks as follows:

(4) $$y=15.1-0.175*t+2.3m_{1}-2.1m_{2}-4.3m_{3}-0.9m_{4}+0.7m_{5}-0.1m_{6}+5m_{7}+2.2m_{8}+0.8m_{9}+0.1m_{10}-1.7m_{11}-1.7m_{12}$$

**Fig. 10:**
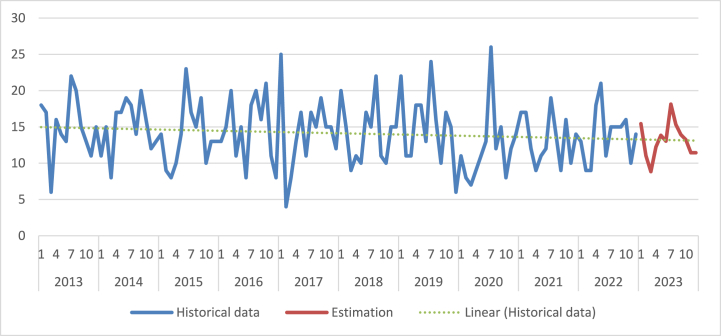
– Visualisation of the historical and estimated numbers of emergency events at level crossings in the Czech Republic.

Estimation of values in the following months of the year of 2023 is obtained by using *t* = 11 (estimating eleventh element when using 10 element input array) to the regression coefficient and choosing *m* = 1 for the corresponding month of the regression coefficient in the equation. The trend estimate for the following months is shown in Table 10 and Fig. 10. In total for 2023, the estimate of the number of emergency events is estimated to 158. This value is equal to the value estimated using the annual data in the first regression analysis.

**Table 10:** – Estimation of the number of emergency events at level crossings in the Czech Republic in months of the year of 2023.

Year	Corresponding *t*	Month	Number of emergency events (estimation)
2023	11	1	15
		2	11
		3	9
		4	12
		5	14
		6	13
		7	18
		8	15
		9	14
		10	13
		11	11
		12	11

## 4 Discussion

Linear regression using annual data was demonstrated in collisions at level crossings equipped with a warning cross. Surprisingly, linear regression was refuted in the case of emergency events at level crossings that are equipped with light and sound signalling equipment with barriers.

The output of the second analysis using time series (seasonality, monthly data) show that the highest number of emergency events take place usually in summer (June, July, August) and in January. In contrary, the lowest number of emergency events is usually at the end of winter (February, March). Computing estimation of the total number of emergency events for 2023 using annual or monthly data results in the same number (158) as expected.

This study has certain limitations that should be acknowledged. The selected 10-year period may be regarded as a relatively small sample size, which may not sufficiently meet the assumptions required for linear regression analysis. This is based on the fact that official resources available provide only a limited history of detailed data pertinent to this research. Despite these constraints, the authors endeavored to identify potential trends and patterns within the data, contributing to a preliminary understanding of the subject matter.

Another limitation pertains to the scope of the research. The findings and conclusions drawn from this study cannot be generalized or directly applied beyond the context of the Czech Republic. However, the data and calculations presented in this article could potentially address similar questions regarding the safety of level crossings and corresponding trends in other countries. Furthermore, many of the proposals discussed in this section may be relevant and applicable in different countries.

Based on the results of the emergency events investigation, the Rail Inspection has issued several safety recommendations in the past. The most important ones are.

• A system of numbering of level crossings (since 2009), which enables their unambiguous identification on the back side of the warning cross of each level crossing in the Czech Republic. In the event of an accident or suspicion of incorrect operation of the signalling equipment, it is necessary to report the level crossing number to the emergency line [11].
• The Rail Inspection has been recommending securing railway crossings with light warning signals and barriers for a long time, as it is the safest way of securing road and rail crossings according to the number and consequences of emergency events.
• In 2020, the warning crosses, including any vertical traffic signs “Stop, give way!”, were provided with a retroreflective fluorescent yellow-green background, thus increasing the visibility of the level crossing markings for road users [11].
• Moreover, the Rail Inspection continues to recommend the previously issued safety recommendations aimed at increasing safety at level crossings.

One of the other ways to minimize the number of emergency events may be more frequent installation of technical safety equipment that would prevent human error when driving a railway vehicle. This ETCS (European Train Control System) would ensure higher operational safety, while gradually replacing many different, obsolete, non-cooperative systems with which domestic railway lines are equipped [22]. At the same time, it is also necessary to equip traction vehicles with this system. However, it will be necessary to spend a lot of money to implement this measure, as equipping one locomotive with the ETCS system costs about 10 million CZK. Railway protection with this system is just as expensive. For example, the section from Pilsen to Cheb, which was about 100 km long, cost the Railway Administration about a billion CZK [22].

As this issue is very topical, the authors propose other suitable measures that could lead to a reduction in collisions of road users at different types of level crossings with railway sets.

• Gradually continue to equip level crossing safety installations with an emphasis on light and sound safety installations equipped with barriers at level crossings equipped only with a warning cross.
• Cancel little-used level crossings.
• Railway crossings equipped with light and sound signalling need to be equipped also with barriers that will already have built-in red LED lights [23], which will be triggered together with a pair of red lights placed on the warning sign.
• Build a grade-separated crossing of busy railway crossings despite the financial demands.
• In the cases of two half-barriers on each side of the road at a level crossing, equip these crossings with sequential folding of barriers.
• Continue to install so-called smart cameras at all level crossings [24], which would send offences directly to law enforcement authorities and punish these drivers with significant financial penalties.
• Ensure the installation of warning lights in the road in front of the level crossing itself to create a psychological barrier for road users. These lights would switch on together with a pair of red warning lights located on the warning sign. A cheaper solution may be spraying of horizontal traffic signs in the form of a continuous transverse line.
• Improve the visibility of level crossings equipped only with a warning cross by means of traffic signs made of retroreflective and fluorescent materials [25].
• Install a laser system [26] that checks the level crossing location when the barriers are activated and in the event of an obstacle detection, the signalling equipment is triggered to stop railway sets approaching the level crossing.
• Remove obstacles and barriers near level crossings to ensure drivers have adequate visibility before crossing.
• Increase penalties for road users who enter level crossings at prohibited times and, in this context, increase the involvement of law enforcement authorities in this process.
• Continue the positive campaign in the mass media shedding light on the undisciplined behaviour of road users at the level crossing.

There is also a toolbox aimed at higher level crossings safety [37] (as one of the results of a European project called SAFER-LC [35]) already mentioned at the end of the introduction of this article.

## 5 Conclusion

Due attention is paid to safety on railway lines with an emphasis on level crossings. Despite this, many human lives are lost at level crossings every year (especially in summer) and considerable damage is caused to the property of road users or entities operating these level crossings. From the point of view of long-term statistics, the most tragic consequences of collisions are at level crossings secured by light warning signalling without barriers. The Railway Inspectorate has long recommended that as many level crossings secured by light signalling equipment as possible should be supplemented with barriers. This method of securing the level crossing of the road and the railway line (compared to the installation of warning crosses with light signalling) seems to be the least risky for road and rail transport in the long term. The authors of the article agree with this conclusion. The Annual Reports of the Rail Inspection in the Czech Republic show that killed and injured persons at level crossings are mostly unruly pedestrians or persons acting with suicidal intent, especially on the busiest lines or on lines with higher line speed. In addition to these pedestrians, it is also possible to encounter undisciplined drivers who do not respect the traffic rules concerning the way of safely crossing the level crossing. Using linear regression methods on both annual and monthly datasets, the authors of the article pointed out trends of emergency events on railway lines with an emphasis on level crossings, including a summary of proposals for their minimization.

## CRediT authorship contribution statement

**Jan Strohmandl:** Writing – original draft, Conceptualization. **Pavel Tomášek:** Writing – review & editing, Visualization, Formal analysis. **Miroslav Tomek:** Writing – original draft, Resources. **Dušan Vičar:** Supervision, Methodology. **Jakub Rak:** Validation, Methodology. **Roman Novák:** Resources, Investigation.

## Data availability statement

Data included in article are fully referenced in article (public sources).

## Declaration of competing interest

The authors declare that they have no known competing financial interests or personal relationships that could have appeared to influence the work reported in this paper.
